# The relationship between religious/spiritual well-being, psychiatric symptoms and addictive behaviors among young adults during the COVID-19-pandemic

**DOI:** 10.3389/fpsyg.2022.942149

**Published:** 2022-09-12

**Authors:** Xenia D. Vuzic, Pauline L. Burkart, Magdalena Wenzl, Jürgen Fuchshuber, Human-Friedrich Unterrainer

**Affiliations:** ^1^Institute of Psychology, University of Graz, Graz, Austria; ^2^CIAR: Center for Integrative Addiction Research, Grüner Kreis Society, Vienna, Austria; ^3^Department of Philosophy, University of Vienna, Vienna, Austria; ^4^Department of Psychiatry and Psychotherapeutic Medicine, Medical University Graz, Graz, Austria; ^5^Department of Religious Studies, University of Vienna, Vienna, Austria

**Keywords:** religious/spiritual well-being, COVID-19, mental health, addictive behavior, young adults

## Abstract

**Background:**

It is becoming increasingly apparent that the COVID-19 pandemic not only poses risks to physical health, but that it also might lead to a global mental health crisis, making the exploration of protective factors for mental well-being highly relevant. The present study seeks to investigate religious/spiritual well-being (RSWB) as a potential protective factor with regard to psychiatric symptom burden and addictive behavior.

**Materials and Methods:**

The data was collected by conducting an online survey in the interim period between two national lockdowns with young adults (*N* = 306; age: 18–35) in Austria. The primary study variables were assessed through the Brief Symptom Inventory 18 (BSI-18; psychiatric symptom load), the Alcohol, Smoking and Substance Involvement Screening Test (ASSIST; addictive behavior/addiction risk) and the Multidimensional Inventory for Religious/Spiritual Well-Being short version (MI-RSWB 12), with its sub-dimensions Hope (HO), Forgiveness (FO), General Religiosity (GR), and Connectedness (CO).

**Results:**

We observed HO and FO as substantial negative predictors of psychiatric symptom burden. With regard to addictive behavior, HO in particular but also GR seem to have a protective function. Furthermore, we found positive connections between CO, psychiatric symptom burden, and addictive behavior.

**Conclusion:**

In line with our assumptions, HO, FO and to a minor extent GR were confirmed as negative predictors regarding psychiatric symptom burden or addictive behavior in young adults, coping with the psychological threat of COVID-19 pandemic. These dimensions might be further considered as potential resources in clinical treatment. However, the positive prediction of mental illness parameters by increased feelings of CO could also be interpreted as an expression of exhaustion and alienation from the real world.

## Introduction

The outbreak of the COVID-19 pandemic has led to a worldwide collective state of emergency within a short period of time. All of a sudden, the global population was faced with new challenges concerning nearly all aspects of everyday life without the possibility of falling back on contemporary experience. It became apparent that the pandemic not only poses a risk to our physical health, but that the necessary socio-political steps taken might have a severe impact on our mental health. The World Health Organization (2020) warned already at the beginning of the pandemic that self-isolation and quarantine could lead to an increase in anxiety, depression and addictive behavior. Recent research confirms these concerns on a global scale: in many countries severely affected by the pandemic, an increase in psychiatric symptoms and psycho-social problems was observed (Gavin et al., 2020; González-Sanguino et al., 2020; Kumar and Nayar, 2020; Pieh et al., 2020; Vindegaard and Benros, 2020).

It is expected that the pandemic’s psycho-social consequences will be unequally distributed and that certain groups of the population will suffer more than others: especially people who were already facing difficulties before the crisis, such as the ones being exposed to threatening situations (e.g., unemployment, domestic violence, marginalization, homelessness; Blustein and Guarino, 2020; Lima et al., 2020; Usher et al., 2020), single parents, especially single mothers (Alon et al., 2020), and people with pre-existing mental health conditions or addiction problems (Vindegaard and Benros, 2020). Nevertheless, even people without prior significant difficulties are affected since the pandemic might interfere with their academic, occupational and interpersonal functioning (Charles et al., 2021). The situation can provoke feelings of uncertainty, ambiguity and loss of control, causing internalizing symptoms leading to states of anxiety and depression (Kujawa et al., 2020). Brooks et al. (2020) point out that quarantine is associated with increased psychological distress, depression and anxiety. Additionally, many people report a constant fear of getting infected or unknowingly infecting other people with the virus, of loved ones getting ill (Mertens et al., 2020), or being socially stigmatized due to a COVID-19 diagnosis (Sotgiu and Dobler, 2020).

In several studies, Pieh et al. (2020) examined the Austrian population’s mental health since the start of the COVID-19 pandemic, demonstrating a significant increase in depression, anxiety and sleep problems, especially among people without prior psychiatric diagnoses. In correspondence to previous work, young adults (age range 20–30) displayed the highest amounts of psychiatric distress compared to other age groups. Although the risk for young adults (without pre-existing health conditions) to experience a severe COVID-19 course is relatively low, young adults have shown strikingly high stress levels since the onset of the crisis (Twenge et al., 2019; Shanahan et al., 2020; Charles et al., 2021). According to Pieh et al. (2020), 50 % of the young adults who took part in their online survey reported depressive symptoms in the second half of 2020—an increase of 20 percent compared to the year before the pandemic. For most individuals, young adulthood represents a formative period, involving transitions and changes related to academic or professional development, social and romantic relationships, or moving out of the parental home (Arnett, 2000). Transitions and changes that are generally known to be stressful (Twenge et al., 2019) could be exacerbated by the pandemic’s triggered stressors or interruptions (Shanahan et al., 2020).

Along with the increase of depression, anxiety, or feelings of loneliness, also the risk of development of an addictive disease rises (Swendsen and Merikangas, 2000; Boden and Fergusson, 2011; Charles et al., 2021). Especially in times of crisis, psychoactive substances could be misused as a means to escape from a stressful reality. Thus, constant brooding, fewer positive distractions or obligations and a lack of social support can trigger addictive behavior (Charles et al., 2021). A representative online study on behalf of the Austrian Federal Ministry of Social Affairs, Health, Care and Consumer Protection surveying 6,000 Austrians regarding their consumption of psychotropic drugs (alcohol, nicotine, cannabis, sleeping pills and tranquilizers) during the first lockdown showed that for the majority, in terms of quantity, it remained the same. Only a minority in each case reported having consumed less or more. However, a pattern of vulnerable individuals can be observed regarding increased consumption. Women were much more likely to report increased substance use than men, especially in regard to sleeping pills and tranquilizers (Kompetenzzentrum Sucht, 2020).

According to the biopsychosocial model of health and disease, mental health can be understood as a multidimensional process influenced by biological, psychological and social factors interacting with each other. It states that mental health not only implies the absence of illness—it is rather reflected in a person’s self-regulating ability to cope with stressors and to use one’s resources adequately (Engel, 1979; Borell-Carrió et al., 2004). Dealing with everyday life during a pandemic requires high levels of mental strength, adequate coping strategies and stress resilience, especially when psychological or medical help services are less accessible.

An essential factor of mental health that has received more attention over the past decade is the protective function of religion or spirituality as a way of coping with stress or illness (Unterrainer et al., 2013, 2014). In this context, complementary to the biopsychosocial approach, the concept of religious/spiritual well-being (RSWB) was developed as a further relevant component to mental health and subjective well-being (Ellison, 1983; Saad et al., 2017). Unterrainer et al. (2011, p. 117) define RSWB as “…the ability to experience the meaning and significance of one’s existence through a sense of connection with oneself, others, or a power greater than oneself” within a transcendental (religious/spiritual) and immanent (biopsychosocial) space. While the immanent dimension encompasses grounded aspects of spirituality such as forgiveness and hope for a better life, the transcendent dimension is understood to include spiritual/religious concepts such as connectedness, prospect of life after death and general religiosity. Thus, RSWB includes both a psycho-social dimension (immanent) and a more religious/spiritual dimension (transcendent) which together form an essential tool that aims to integrate and enhance physical, psycho-emotional and social aspects of health (c.f. Ellison, 1983).

Substantial evidence points to the salutogenic impact of religious and spiritual factors (R/S) on psychological well-being, coping and resilience (Smith et al., 2003; Ozawa et al., 2017). Correspondingly, a wide range of positive effects of RSWB on physical and mental health was observed, leading to higher levels of subjective life satisfaction, hope, optimism and lower rates of anxiety, depression, and substance abuse (Koenig, 2012; Lucchetti et al., 2020; Unterrainer, 2022). Pardini et al. (2000) found in their study focused on inpatient drug withdrawal that higher levels of R/S were associated with a more optimistic life orientation, greater social support, higher resilience to stress and lower anxiety and ultimately predicted a more positive therapy outcome. Based on such findings, R/S became increasingly important in clinical and inpatient settings, being integrated into diagnosing, treating and rehabilitating patients with psychiatric symptoms and/or addiction problems (Shirzad et al., 2020; Fuchshuber and Unterrainer, 2021). Coppola et al. (2021) emphasize R/S as being closely related to more adequate coping, specifically concerning the function of processing stressful events, such as the consequences of a global pandemic. Correspondingly, R/S were associated with a better ability to cope with serious illness or isolation (Sharma et al., 2017; Le et al., 2019). In line with these findings, it is expected that especially in times of a global pandemic, RSWB would have protective functions preventing psychiatric symptoms and addictive behavior. Lastly, it must be mentioned here that also negative or no correlations can be found in the literature between religion, spirituality and various parameters of physical and mental health. To avoid over-interpretation of the results, this important point should always be kept in mind (for a critical review see, e.g., Koenig and Larson, 2001).

### Study aims

In this study, it is aimed to investigate the relationship between RSWB dimensions, psychiatric symptom load and addictive behavior among young adults during the COVID-19 pandemic. RSWB is expected to negatively predict the extent of psychiatric symptom burden and the risk of addictive behavior. Furthermore, we expect a positive connection between psychiatric symptoms (somatization, depression, and anxiety) and addictive behavior.

## Materials and methods

### Sample and procedure

The investigated sample consisted of 306 (72.5% female) German-speaking individuals. Participation was voluntary and anonymous. The subjects were recruited *via* the e-mail distribution list of the University of Graz and the data was acquired *via* the online survey platform Lime Survey©. After informed consent was given, participants answered demographic questions (e.g., age, sex, education level) followed by standardized questionnaires. Participants aged between 18 and 35 years who completed the whole questionnaire were included. Accordingly, 160 participants of the total sample (*N* = 466) had to be excluded. Ethical approval was granted by the Ethics Committee of the University of Graz, Austria. The study was carried out from October 2020 to November 2020, between two national lockdowns in Austria.

### Psychometric assessment

The *Multidimensional Inventory for Religious/Spiritual Well-Being short version* (MI-RSWB 12; Unterrainer and Kapfhammer, 2014; Fuchshuber and Unterrainer, 2021) is a self-report measure that assesses different dimensions of spiritual and religious well-being. It is the short version of the MI-RSWB 48 (Unterrainer et al., 2010, 2014) and consists of 12 items that are rated on a 6-point Likert scale ranging from 1 (“strongly disagree”) to 6 (“strongly agree”). These items can be grouped into four subscales (3 items per subscale: “General Religiosity” [GR], “Connectedness” [CO], “Forgiveness” [FO] and “Hope” [HO]). The addition of all items results in a total score, defined as “Religious/Spiritual Well-Being” (RSWB). Fuchshuber and Unterrainer (2021) report excellent psychometric properties for the scale (see also Fuchshuber and Unterrainer, 2021, supplementary materials; for the list of items together with a short manual).

*The Brief Symptom Inventory 18* (BSI-18; Derogatis, 2001) is a self-report measure to capture symptoms of somatization, depression and anxiety over the past 7 days. It is the abbreviated version of the Brief-Symptom-Inventory-53 (BSI-53, Derogatis and Melisaratos, 1983), which in turn is the short form of the Symptom-Checklist (SCL 90-R; Derogatis and Clear, 1977). The 18 items, rated on a 5-point Likert scale ranging from 0 (“absolutely not”) to 4 (“very strong”), sum up to the three subscales: Depression, Anxiety and Somatization. A total score “Global Severity Index” (GSI) can be generated by adding the scores of every item, reflecting the current amount of general psychological distress. Higher scores represent more significant distress. For the present study, the German version of the BSI-18 by Spitzer et al. (2011) was applied.

*The Alcohol, Smoking and Substance Involvement Screening Test* (ASSIST; Heslop et al., 2013) is a standardized interview that is applied to assess risky or problematic substance consumption. It was developed by the World Health Organization and is intended to serve as a screening tool, mainly in primary care settings, where harmful drug use often goes undetected. It measures the use of substances and its associated problems over the last 3 months as well as over one’s lifetime, regarding the consumption of tobacco, alcohol, cannabis, cocaine, amphetamines, inhalants, sedatives, hallucinogens, opioids and “other drugs.” Questions 2–5 assess the “frequency of drug use,” “craving to use the drug,” “problems because of drug use” and “failed expectations” and are rated on a 7-point Likert scale ranging from 0 (“never”) to 6 (“daily or almost daily”). Questions 6, 7, and 8 measure “expressed concerns by relatives or friends,” “failed attempts to cut down drug use” and “drug injection” and are rated on a 3-point scale (0 = “no, never”; 3 = “yes, but not in the past 3 months”; 6 = “yes, in the past 3 months”). Drug-specific sub-scores are created, representing the level of addiction risk for each substance. For this study, an overall score was calculated by summing up the drug-specific symptom scores.

Furthermore, a list of socio-demographic variables was included into the test-form. These were age, gender, educational qualification, marital status and religious affiliation.

### Statistical analysis

SPSS 27.0 was used for data management, descriptive statistics, bivariate correlations, and regression modelling. Due to the explorative character of the study, α-level was set to 0.05.

## Results

### Sample characteristics

The investigated sample consisted of 306 (72.5% females) German-speaking young adults ranging in age from 18 to 35 years (*M* = 22.2; *SD* = 3.8). Most participants stated the general qualification [Abitur, Matura] for university entrance (*n* = 249; 81.4%) or a university degree (*n* = 53, 17.3%) as their highest educational qualification. Regarding their religious affiliation, 119 (38.9%) identified themselves as religious, while 159 (52%) stated not being religiously affiliated and 28 (9.2%) decided not to answer this question. In terms of their current relationship status, 162 (52.9%) participants stated they were single, 135 (44.1%) to be in a relationship, three being married (1%), one (0.3%) being divorced and five (1.6%) did not give any information. In terms of their consumption patterns regarding psychoactive substances during the pandemic, 62 (20.3%) participants reported having used more alcohol than usual, and 31 (10.1%) reported having used more drugs (other than alcohol) during the period of the first national lockdown in Austria (mid-March to the end of May 2020).

As a first step, Confirmatory Factor Analysis (CFA) for the MI-RSB 12 revealed acceptable model fits for both a general factor/bifactor model [*χ*^2^/df = 1.74; RMSEA = 0.05 (90% CI: 0.03, 0.07); CFI = 0.98; NFI = 0.95; TLI = 0.96], as well as a model which assessed every subscale as an independent factor [*χ*^2^/df = 1.96; RMSEA = 0.06 (90% CI: 0.04, 0.07); CFI = 0.96; NFI = 0.93; TLI = 0.95]. These results are generally in line with previous estimations by Fuchshuber and Unterrainer (2021).

### Bivariate correlations between the MI-RSWB 12 subscales, BSI-18 and ASSIST

As a further step, descriptive statistics (means and standard deviations) and Pearson’s correlations for the scores of the main study variables BSI-18, ASSIST and MI-RSWB 12 were calculated. As presented in Table 1, the GSI of the BSI shows significant negative correlations with the MI-RWSB subscales General Religiosity (*r* = −0.123, *p* < 0.05), Forgiveness (*r* = −0.264, *p* < 0.01) and Hope (*r* = −0.445, *p* < 0.01). The ASSIST total score was found to be significantly positively correlated with the MI-RSWB subscale Connectedness (*r* = 0.129, *p* < 0.05) and negatively correlated with the MI-RSWB subscales Forgiveness (*r* = −0.141, *p* < 0.05) and Hope (*r* = −0.186, *p* < 0.01). Furthermore, BSI-GSI and the ASSIST total score show a significant correlation (*r* = 0.276, *p* < 0.01).

**Table 1:** Means, standard deviations, and bivariate correlations between the study variables of MI-RSWB, BSI, and ASSIST.

Variables	*M*	*SD*	2	3	4	5	6
1. MI-RSWB_GR	6.02	3.90	0.154^**^	0.333^**^	0.496^**^	−0.123^*^	−0.061
2. MI-RSWB_FO	14.31	3.22	–	0.121^*^	−0.090	−0.264^**^	−0.141^*^
3. MI-RSWB_HO	12.84	3.16		–	0.244^**^	−0.445^**^	−0.186^**^
4. MI-RSWB_CO	7.49	4.06			–	0.071	0.129^*^
5. BSI_GSI	13.89	10.39				–	0.276^**^
6. ASSIST_Total	24.40	31.28					–

*N* = 306. MI-RSWB, Multidimensional Inventory for Religious/Spiritual Well-Being short version (GR, General Religiosity; FO, Forgiveness; HO, Hope; CO, Connectedness); BSI, Brief Symptom Inventory 18 (GSI, Global Severity Index); ASSIST, Alcohol, Smoking and Substance Involvement Screening Test.

^*^p < 0.05; ^**^p < 0.01.

### Regression analyses: MI-RSWB 12 subscales on BSI-18 and ASSIST

The central research question of the present study was whether RSWB positively affects psychiatric symptom burden and addictive behavior in terms of a protective factor. For this purpose, two multiple linear regressions were conducted. Based on theoretical considerations, the MI-RSWB 12 subscales Hope, Forgiveness, Connectedness and General Religiosity were considered predictors for the respective models. All requirements for conducting a multiple linear regression analysis were met in both models after excluding outliers (SDR values <−3 or >3).

In the first regression model, the aim was to determine whether the BSI-GSI can be predicted in a regression model based on the four MI-RWSB subscales (as presented in Table 2). The *R*^2^ for the overall model was 0.29 (adjusted *R*^2^ = 0.28), indicative of a high goodness-of-fit according to Cohen (1992) and statistically significant. The MI-RSWB subscales Hope (*ß* = −0.474, *p* < 0.01) and Forgiveness (*ß* = −0.188, *p* < 0.01) were found to be the strongest significant predictors for the BSI-GSI in the model. Furthermore, the MI-RSWB subscale Connectedness (*ß* = 0.167, *p* < 0.01) also exhibits significant predictive power for the obtained BSI-GSI score, but in terms of a positive correlation (higher Connectedness scores predict higher BSI-GSI scores). The MI-RWSB subscale General Religiosity (*ß* = −0.055, *p* = 0.354) does not represent a significant predictor for the BSI-GSI score.

**Table 2:** Regression model: *BSI-GSI Score* as dependent variable predicted by the four subscales of religious/spiritual well-being.

Variables	*b*	*SE b*	*ß*	*t*	*R*^2^ adj
1. MI-RSWB_GR	−0.142	0.153	−0.055	−0.928	0.282
2. MI-RSWB_FO	−0.585	0.157	−0.188	−3.720^**^	
3. MI-RSWB_HO	−1.508	0.166	−0.474	−9.083^**^	
4. MI-RSWB_CO	0.417	0.142	0.167	2.931^**^	

*N* = 303. MI-RSWB, Multidimensional Inventory for Religious/Spiritual Well-Being short version (GR, General Religiosity; FO, Forgiveness; HO, Hope; CO, Connectedness).

^*^p < 0.05; ^**^p < 0.01.

With regard the regression analysis of the MI-RSWB subscales on the total score obtained in the ASSIST, merely 8% of the variance can be elucidated (see Table 3). Thereby, General Religiosity (*ß* = −0.157, *p* < 0.05) and Hope (*ß* = −0.158, *p* < 0.01) represent significant, but still weak predictors in terms of a negative relation (higher scores on the subscales General Religiosity and Hope predict lower ASSIST scores) and Connectedness (*ß* = 0.270, *p* < 0.01) in terms of a positive relation (higher scores on the Connectedness scale predict higher ASSIST scores). Forgiveness (*ß* = −0.078, *p* > 0.05) does not show predictive significance for the ASSIST total score.

**Table 3:** Regression model: ASSIST Total Score as dependent variable predicted by the four subscales of religious/spiritual well-being.

Variables	*b*	*SE b*	*ß*	*t*	*R*^2^ adj
1. MI-RSWB_GR	−0.850	0.367	−0.157	−2.316^*^	0.080
2. MI-RSWB_FO	−0.518	0.379	−0.078	−1.369	
3. MI-RSWB_HO	−1.073	0.404	−0.158	−2.656^**^	
4.MI-RSWB_CO	1.407	0.341	0.270	4.120^**^	

*N* = 301. MI-RSWB, Multidimensional Inventory for Religious/Spiritual Well-Being short version (GR, General Religiosity; FO, Forgiveness; HO, Hope; CO, Connectedness).

^*^p < 0.05; ^**^p < 0.01.

### BSI norm sample comparison

In order to compare the BSI Scores of our sample to pre-pandemic data of a representative non-clinical German speaking sample (*N* = 293; age range: 25–35; Spitzer et al., 2011), two-tailed one-sample t-tests were conducted. It revealed that both the mean GSI score (*M* = 13.90), *t*(305) = 18.25, *p* < 0.001 and the mean scores of the subscales Anxiety (*M* = 4.56), *t*(305) = 15.88, *p* < 0.001; Depression (*M* = 5.78), *t*(305) = 15.60, *p* < 0.001; and Somatization (*M* = 13.90), *t*(305) = 14.44, *p* < 0.001 were significantly higher than the average values of the norm sample (GSI: *M* = 3.05, *SD* = 5.7; SOM: *M* = 0.70, *SD* = 1.8; DEP: *M* = 1.27, *SD* = 2.5; ANX: *M* = 1.09, *SD* = 2.1).

## Discussion

This study, carried out in-between two national lockdowns, aimed to further understand mental health risks posed by the consequences of the COVID-19 pandemic and in particular to identify the potential salutogenic effect of RSWB dimensions on mental health. As observed in previous research, the COVID-19 life circumstances turned everyday life for many individuals into an unusually stressful time affecting their psychological well-being severely (Vindegaard and Benros, 2020). Concerns that the pandemic will lead to a global mental health crisis are increasingly coming to the fore, which is why it is of vital importance to shed light on possible protective factors (Dong and Bouey, 2020; Vindegaard and Benros, 2020). In line with this, different dimensions of religiosity/spirituality might be considered as potential resources.

In the present study we were able to confirm several findings from previous research, as a salutogenic function especially of the RSWB dimension Hope and to some extent Forgiveness concerning mental health during the global COVID-19 pandemic could be observed (*cf.* Hiebler-Ragger et al., 2016). Therefore, the obtained results demonstrate a positive impact of RSWB on different aspects of mental health: in particular, high levels of Hope might help to ease or avoid symptoms of anxiety, somatization and/or depression that otherwise could have arisen. Unterrainer (2022, p. 36) defines this dimension as “hope for a more fulfilling life in the future or that things will change for the better.” Especially in demanding and stressful times of unprecedented uncertainty, hope as a partial attribute of psychological well-being can function as a source of comfort, serenity and confidence. Hope is often reflected in an active minimization of worries or concerns, the ability to look confidently to the future and recall positive memories appropriately (Wnuk and Marcinkowski, 2014). Furthermore, we observed some beneficial potential of the Forgiveness dimension. This dimension has been described “as the ability to forgive oneself or other people or to resign oneself to the things that have gone wrong” (Unterrainer, 2022, p. 36). Consequently, an increased amount of Forgiveness might contribute to the ability to deal with the pandemic more mindfully by avoiding negative feelings towards others (such as blaming or grudging) and rejecting a victim role in a self-determined way (Baumeister et al., 1998). Thus, scoring high on the Forgiveness and Hope scale could be reflected in the accessibility of healthy coping mechanisms that might prevent further accentuation of negative mental health aspects. It is of note that both Hope and Forgiveness have been nominated as being the immanent space of perception, which represent more grounded and more biopsychosocial aspects of the RSWB concept. A bit in contrast to our assumptions, an increased amount of Connectedness was found to predict a higher severity of psychiatric symptoms. A possible interpretation might be that for individuals who show a strong feeling of the relatedness to their environment (including the universe etc.), such a drastic development might have specific severe impact. On the one hand, they may be affected by the collective suffering and, on the other hand, a feeling that wrongdoings might have caused these circumstances might emerge and obstruct healthy coping.

Although not as strong as for psychiatric symptom load, RSWB also showed predictive power for addiction risk, as higher levels of Hope and General Religiosity were negatively related with the risk of developing substance abuse or addiction. Unterrainer (2022, p. 36) defines General Religiosity as “a religious belief in the traditional sense, and/or being affiliated to a religious community.” A religious or spiritual sense of belonging in the traditional sense might discourage individuals from excessive substance use, particularly based on their beliefs or principles (*c.f.* Cook, 2004; Geppert et al., 2007). As noted earlier, RSWB (especially Hope) can activate healthy coping mechanisms in challenging situations. However, if RSWB turns out to be very low or impaired and subsequently coping mechanisms are lacking, psychoactive substances may be abused as a maladaptive replacement strategy. Contrary to our assumptions the FO scale failed to provide influence on the ASSIST (see Table 3).

Furthermore, a positive connection between psychiatric symptom burden and addictive behavior was confirmed. Thereby, psychiatric symptoms such as anxiety, somatization and depression might have a delayed effect on individuals’ addictive behavior. Nevertheless, it can be assumed that pre-existing psychiatric symptoms increase the risk of developing a substance abuse disorder (Swendsen and Merikangas, 2000).

## Limitations and future perspectives

However, there are several limitations to be noted. The homogeneity of the sample definitely limits its representativity; the gender ratio is very one-sided due to a clear majority of female participants, and the age range is limited to young adults. For this reason, rather limited statements can be made about possible gender or age differences, which could be of interest regarding the topic. The degree of socio-economic circumstances is also clearly limited by an entirely academic sample. Due to a voluntary, “at home” online survey format, there is still a risk of unverifiable losses in reliability. It needs to be considered that a sample with a more diverse background accounting for more at-risk participants might have impacted the results. Longitudinal studies and a more focused investigation of COVID-19 specific variables are needed in order to be able to make reliable statements about the actual impact and consequences of the COVID-19 pandemic on the population’s mental health. It might also be of great interest to investigate deeper the matter of RSWB in relation to the consequences of the pandemic and to evaluate specific religious/spiritual interventions. Although some RSWB dimensions were shown to have a beneficial influence on mental well-being, there are certainly various other salutogenic factors to be highlighted and promoted in the context of the pandemic. Therefore, the need to identify and highlight further beneficial variables which directly influence or mediate/moderate the development of mental health seems highly relevant. For instance, further work could also pay more attention to the resilience factor and distinguish between individuals who are able to withstand the consequences of the pandemic and those who increasingly need to resort to other forms of coping (e.g., religiosity) to manage the negative experiences. Restrictively, it should be also noted that dimensions such as hope can be discussed in a religious/spiritual context, but this need not be the case. As part of spiritual well-being, hope can be considered as a facet of existential well-being (see Ellison, 1983, for further discussion), but hope is also discussed independently as part of psychological well-being (Snyder, 2004). Correspondingly, it should be mentioned that the term “faith” (in Item 1) can be understood or interpreted in different ways, spiritual and/or secular. However, previous studies have shown that the item fits very coherently into the General Religiosity (GR) scale and that this dimension can be assessed highly reliably by means of the scale (see Fuchshuber and Unterrainer, 2021).

## Conclusion

Especially in times like these, it is of great importance to develop protective factors for mental health, as they can be both scientifically and practically beneficial. Not only does the data suggest connections between mental well-being and spiritual well-being, but the inclusion of a spiritual dimension would consequently help to expand the body of knowledge in mental health research and provide a more thorough understanding of human well-being in general. Understanding risk and protective factors can facilitate dealing with many pandemic-related health issues in an appropriate and preventive manner. Especially during challenging times when access to mental health assistance is severely limited, finding practices that promote immanent spiritual growth could have positive outcomes. Practices enhancing RSWB that could easily fit in an at-home setting could reduce or even prevent psychiatric symptoms or psycho-social issues. Further research is needed to examine spirituality-based approaches to offset at least some of the negative mental health consequences caused by the pandemic and to diminish COVID-19-related stress in a mindful, positive and resilient way.

## Data availability statement

The raw data supporting the conclusions of this article will be made available by the authors, without undue reservation.

## Ethics statement

The studies involving human participants were reviewed and approved by University of Graz. The patients/participants provided their written informed consent to participate in this study.

## Author contributions

XV, PB and H-FU conceptualized the study. XV and PB collected the data, XV analyzed and interpreted the data. XV and H-FU drafted the manuscript. JF, MW, and H-FU critically reviewed it. All authors contributed to the article and approved the submitted version.

## Conflict of interest

The authors declare that the research was conducted in the absence of any commercial or financial incentive that could be construed as a potential conflict of interest.

## Publisher’s note

All claims expressed in this article are solely those of the authors and do not necessarily represent those of their affiliated organizations, or those of the publisher, the editors and the reviewers. Any product that may be evaluated in this article, or claim that may be made by its manufacturer, is not guaranteed or endorsed by the publisher.
